# Crystal structure and Hirshfeld surface analysis of 3,3′,3′′-[(1,3,5-triazine-2,4,6-tri­yl)tris­(­oxy)]tris­(5,5-di­methyl­cyclo­hex-2-en-1-one)

**DOI:** 10.1107/S2056989018009003

**Published:** 2018-06-28

**Authors:** Zeliha Atioğlu, Mehmet Akkurt, Flavien A. A. Toze, Gunay Z. Mammadova, Humay M. Panahova

**Affiliations:** a İlke Education and Health Foundation, Cappadocia University, Cappadocia Vocational College, The Medical Imaging Techniques Program, 50420 Mustafapaşa, Ürgüp, Nevşehir, Turkey; b Department of Physics, Faculty of Sciences, Erciyes University, 38039 Kayseri, Turkey; c Department of Chemistry, Faculty of Sciences, University of Douala, PO Box 24157, Douala, Republic of Cameroon; d Organic Chemistry Department, Baku State University, Z. Xalilov Str. 23, Az 1148 Baku, Azerbaijan; e State Economic University of Azerbaijan, Istiqlaliyyat St, 6, AZ1001, Baku, Azerbaijan

**Keywords:** crystal structure, 1,3,5-triazine ring, cyclo­hexenone ring, distorted envelope conformation, hydrogen bonding

## Abstract

The three cyclo­hexenone rings of the title compound, C_27_H_33_N_3_O_6_, adopt slightly distorted envelope conformations, with the C atom bearing two methyl groups as the flap atom in each case. These cyclo­hexenone mean planes form dihedral angles of 87.41 (11), 70.73 (11) and 70.47 (11)° with the 1,3,5-triazine ring, while the dihedral angle between the cyclo­hexenone mean planes are 57.52 (12), 23.75 (12) and 53.21 (12)°. In the crystal, mol­ecules are linked *via* C—H⋯O hydrogen bonds, forming a three-dimensional network.

## Abstract 2

The three cyclo­hexenone rings of the title compound adopt slightly distorted envelope conformations, with the C atom bearing two methyl groups as the flap atom in each case. In the crystal, mol­ecules are linked *via* C—H⋯O hydrogen bonds, forming a three-dimensional network.

## Chemical context   

**Figure 1:**
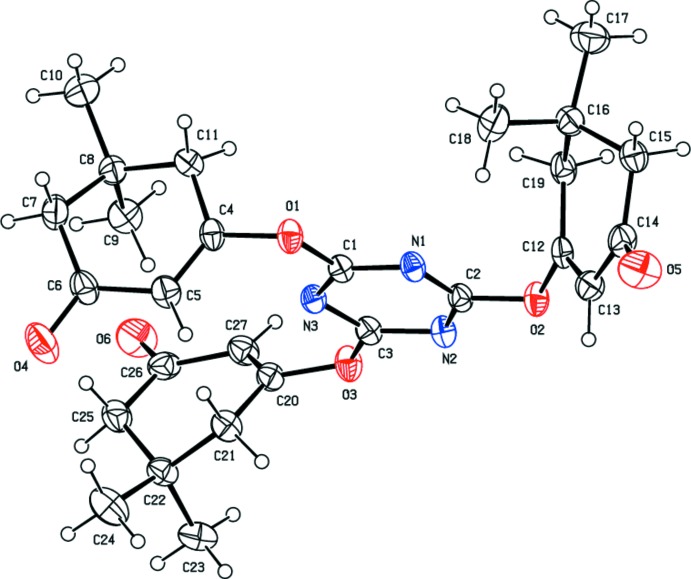
The mol­ecular structure of the title compound. Displacement ellipsoids are drawn at the 30% probability level. H atoms are shown as spheres of arbitrary radius.

β-Diketones are versatile starting materials in the synthesis of organic and coordination compounds (Mahmudov *et al.*, 2017 ▸; Mahmudov & Pombeiro, 2016 ▸). Usually, the active methyl­ene group of β-diketones is a reaction centre in the organic transformations of this class of compounds (Ma *et al.*, 2017*a* ▸,*b* ▸; Gurbanov *et al.*, 2017*a* ▸,*b* ▸, 2018 ▸; Borisova *et al.*, 2018 ▸; Jlassi *et al.*, 2018 ▸). In contrast, there are few reports on the reactivity of β-diketones as *O*-nucleophiles (Yusifov *et al.*, 2013 ▸; Ledenyova *et al.*, 2018 ▸; Vandyshev *et al.*, 2017 ▸; Nasirova *et al.*, 2017 ▸). Herein we found a C—O coupling reaction between cyanuric chloride and dimedone leading to the title compound 3,3′,3′′-[(1,3,5-triazine-2,4,6-tri­yl)tris­(­oxy)]tris­(5,5-di­methyl­cyclo­hex-2-en-1-one) (Fig. 1 ▸).
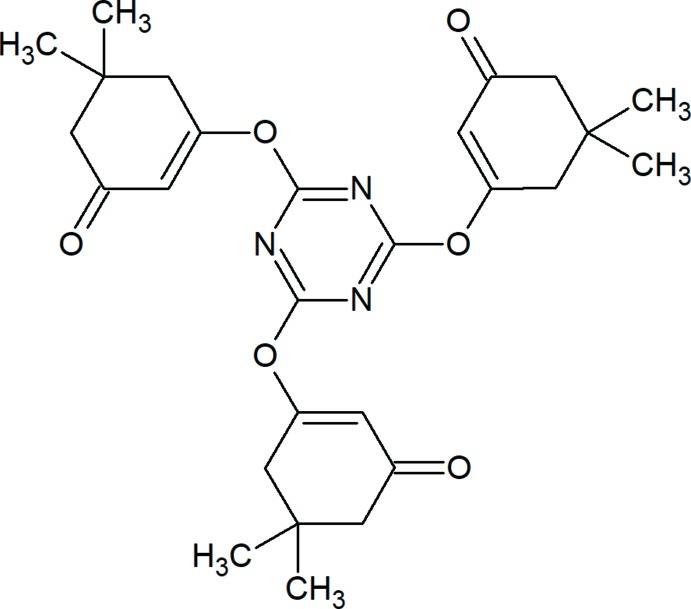


## Structural commentary   

In the title compound, the cyclo­hexenone rings *A* (C4–C8/C11), *B* (C12–C16/C19) and *C* (C20–C22/C25–C27) adopt distorted envelope conformations, with flap atoms C8, C16 and C22, respectively [the puckering parameters are: for *A*, *Q*_T_ = 0.436 (3) Å, θ = 130.8 (4), φ = 43.3 (5)°, for *B*, *Q*_T_ = 0.449 (3) Å, θ = 131.0 (4)°, φ = 46.2 (4)° and for *C*, *Q*_T_ = 0.451 (3) Å, θ = 123.6 (4)°, φ = 298.6 (4)°]. The dihedral angle between the cyclo­hexenone rings are *A*/*B =* 57.52 (12), *A*/*C* = 23.75 (12) and *B*/*C* =53.21 (12)°. The dihedral angle between the 1,3,5-triazine ring (C1/N1/C2/N2/C3/N3) and cyclo­hexenone rings *A*, *B* and *C* are 87.41 (11), 70.73 (11) and 70.47 (11)°, respectively.

The values of the geometric parameters are normal and are comparable to those observed in similar compounds such as 2,2′-[(3-bromo-4-hy­droxy-5-meth­oxy­phen­yl)methyl­idene]bis(3-hy­droxy-5,5-di­methyl­cyclo­hex-2-en-1-one) (Sughanya & Sureshbabu, 2012 ▸) and 3-hy­droxy-2-[(4-hy­droxy-3,5-di­meth­oxy­phen­yl)(2-hy­droxy-4,4-dimethyl-6-oxo­cyclo­hex-1-en-1-yl)meth­yl]-5,5-di­methyl­cyclo­hex-2-en-1-one (Yang *et al.*, 2011 ▸).

## Supra­molecular features   

**Figure 2:**
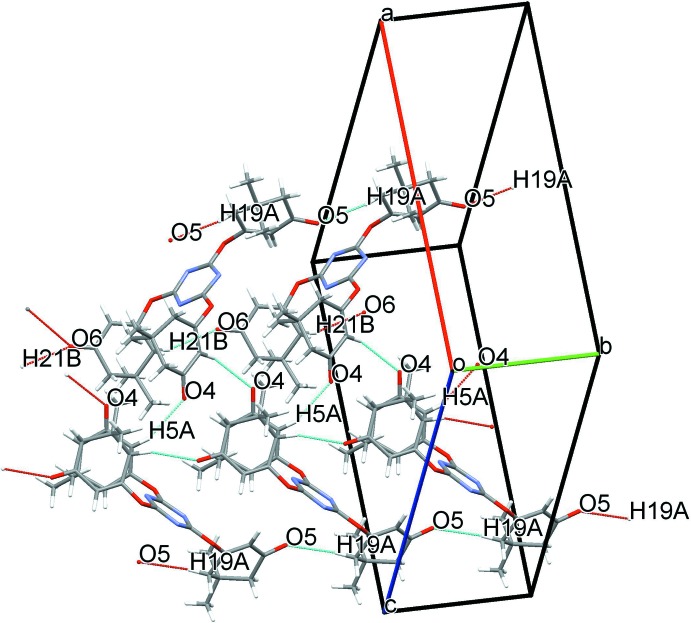
A view of the inter­molecular C—H⋯O hydrogen bonds (Table 1 ▸) in the title compound.

In the crystal, mol­ecules are linked by C—H⋯O hydrogen bonds, forming a three-dimensional network (Table 1 ▸; Fig. 2 ▸). The mol­ecules are further linked by weak C—O⋯π inter­actions between the carbonyl groups, and the centroids (*Cg*1) of the 1,3,5-triazine rings of neighbouring mol­ecules: C14—O5 = 1.213 (3), O5⋯*Cg*11^iii^ = 3.013 (2), C14⋯*Cg*1^iii^ = 3.892 (3) Å, C14—O5⋯*Cg*1^i^ = 129.0 (2)°; C26—O6 = 1.213 (3), O6⋯*Cg*1^ii^ = 3.126 (2), C26⋯*Cg*1^ii^ = 3.899 (3) Å, C26—O6⋯*Cg*1^ii^ = 121.4 (2)°; symmetry codes: (iii) *x*, 1 + *y*, *z*; (ii) *x*, −1 + *y*, *z*]. No C—H⋯π inter­actions or π–π stacking inter­actions are observed in the crystal structure.

## Hiershfeld surface analysis   

**Figure 3:**
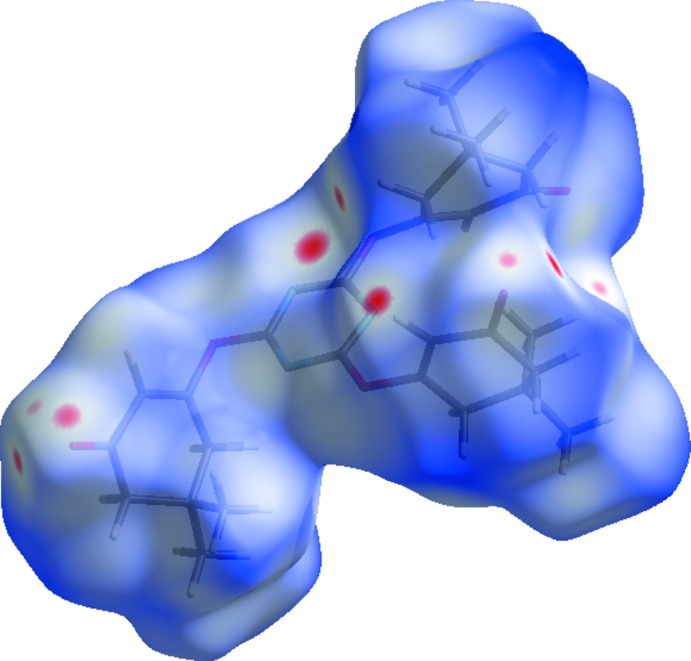
Hirshfeld surface of the title compound mapped over *d*_norm_.

Hirshfeld surfaces and fingerprint plots were generated for the title compound based on the crystallographic information file (CIF) using *CrystalExplorer* (McKinnon *et al.*, 2007 ▸). Hirshfeld surfaces enable the visualization of inter­molecular inter­actions by different colors and color intensity, representing short or long contacts and indicating the relative strength of the inter­actions. Fig. 3 ▸ shows the Hirshfeld surface of the title compound mapped over *d*_norm_(−0.16 to 1.25 a.u.). It is evident from the bright-red spots appearing near the oxygen atoms in this figure that these atoms play a significant role in the mol­ecular packing. The red points, which represent closer contacts and negative *d*_norm_ values on the surface, correspond to the C—H⋯O inter­actions.

**Figure 4:**
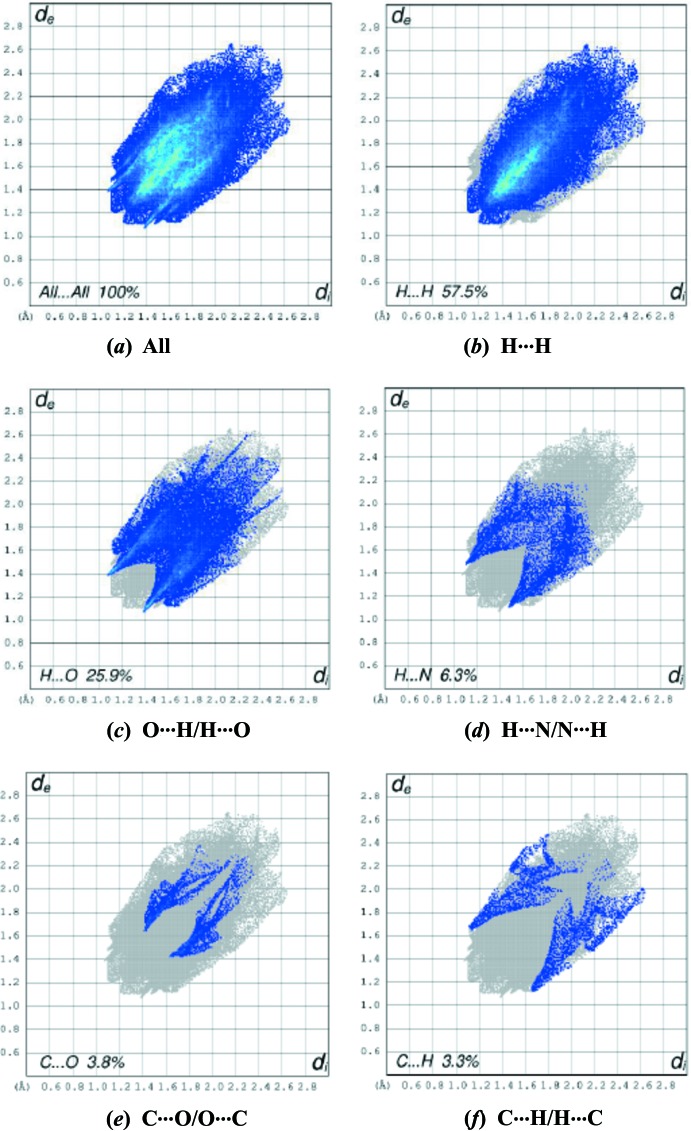
The two-dimensional fingerprint plots of the title compound, showing (*a*) all inter­actions, and delineated into (*b*) H⋯H, (*c*) O⋯H/ H⋯O, (*d*) H⋯N/N⋯H, (*e*) C⋯O/O⋯C and (*f*) C⋯H/H⋯C inter­actions [*d*_e_ and *d*_i_ represent the distances from a point on the Hirshfeld surface to the nearest atoms outside (external) and inside (inter­nal) the surface, respectively].

**Figure 5:**
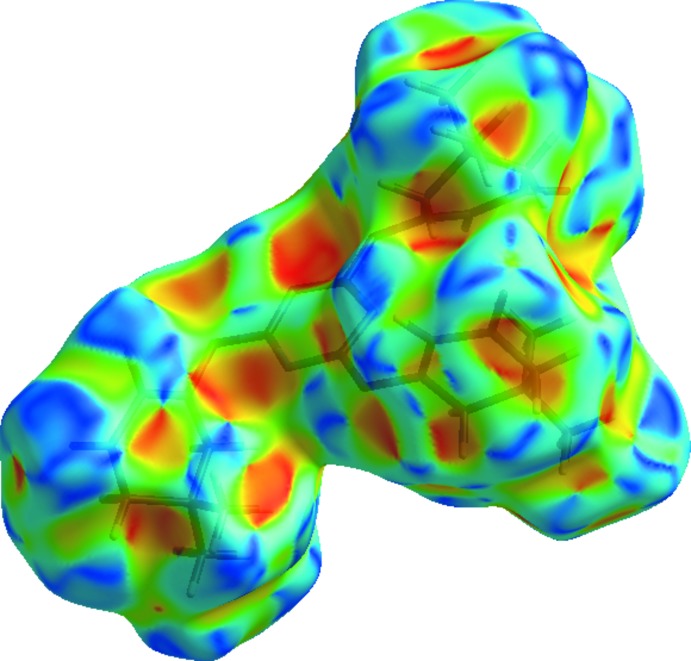
View of the three-dimensional Hirshfeld surface of the title complex plotted over shape-index.

The percentage contributions of various contacts to the total Hirshfeld surface are shown in the two-dimensional fingerprint plots in Fig. 4 ▸. The H⋯H inter­actions appear in the middle of the scattered points in the two-dimensional fingerprint plots with an overall contribution to the Hirshfeld surface of 57.5% (Fig. 4 ▸ *b*). The contribution (25.9%) from the O⋯H/H⋯O contacts, corresponding to C—H⋯O inter­actions, is represented by a pair of sharp spikes characteristic of a strong hydrogen-bonding inter­action (Fig. 4 ▸ *c*). The contribution of the inter­molecular N⋯H/H⋯N contacts to the Hirshfeld surfaces is 6.3% (Fig. 4 ▸ *d*). The small percentage contributions from the other different inter­atomic contacts are as follows: C⋯O/O⋯C (3.8%), C⋯H/H⋯C (3.3%), N⋯O/O⋯N (2.1%), O⋯O (0.9%) and C⋯N/N⋯C (0.2%). The large number of H⋯H, H⋯O/O⋯H and H⋯N/N⋯H inter­actions suggest that van der Waals inter­actions and hydrogen bonding play the major roles in the crystal packing (Hathwar *et al.*, 2015 ▸). The three-dimensional shape-index surface of the title compound is shown in Fig. 5 ▸.

## Synthesis and crystallization   

1.40 g (10 mmol) dimedone was added to 30 mL of an aqueous solution of KOH (0.56 g, 10 mmol) and the solution was stirred for 5 min at room temperature. Cyanuric chloride (0.61 g, 3.3 mmol) was added to this alkali solution of dimedone in 10 portions under stirring for 10 min. After 2 h, the formed white precipitate of the product was filtered off and was recrystallized from methanol. Yield 84% (based on cyanuric chloride), white powder, soluble in DMSO, ethanol and di­methyl­formamide and insoluble in non-polar solvents. Analysis calculated for C_27_H_33_N_3_O_6_ (*M*_r_ = 495.58): C, 65.44; H, 6.71; N, 8.48. Found: C, 65.40; H, 6.65; N, 8.43%. MS (ESI) (positive ion mode): *m*/*z*: 496.73 [*M* + H]^+. 1^H NMR (DMSO-*d^6^*): *δ* 1.01 (18H, 6CH_3_), 1.90 and 2.34 (12H, 6CH_2_), 5.80 (3H, C—H). ^13^C{^1^H} (DMSO-*d^6^*): *δ* 27.80 (6CH_3_), 44.56 (3CH_2_), 48.12 (3CH_2_), 124.31 (3CH), 167.72 (3C=C—O), 176.23 (3C—O) and 196.58 (3C=O).

## Refinement details   

Crystal data, data collection and structure refinement details are summarized in Table 2 ▸. All H atoms were fixed geom­etrically and allowed to ride on the attached non-H atoms, with C —H = 0.93–0.97 Å, and with *U*_iso_(H) = 1.5*U*_eq_(C) for methyl H atoms and 1.2*U*_eq_(C) for all other atoms.

## Supplementary Material

Crystal structure: contains datablock(s) I. DOI: 10.1107/S2056989018009003/zp2030sup1.cif

Structure factors: contains datablock(s) I. DOI: 10.1107/S2056989018009003/zp2030Isup2.hkl

Supporting information file. DOI: 10.1107/S2056989018009003/zp2030Isup3.cml

CCDC reference: 1850649

Additional supporting information:  crystallographic information; 3D view; checkCIF report

## Figures and Tables

**Table 1:** Hydrogen-bond geometry (Å, °)

*D*—H⋯*A*	*D*—H	H⋯*A*	*D*⋯*A*	*D*—H⋯*A*
C5—H5*A*⋯O4^i^	0.93	2.59	3.447 (3)	153
C19—H19*A*⋯O5^ii^	0.97	2.60	3.532 (3)	162
C21—H21*B*⋯O6^iii^	0.97	2.57	3.399 (3)	143

Symmetry codes: (i) 
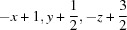
; (ii) 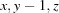; (iii) 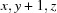.

**Table 2:** Experimental details

Crystal data	
Chemical formula	C_27_H_33_N_3_O_6_
*M*_r_	495.56
Crystal system, space group	Monoclinic, *P*2_1_/*c*
Temperature (K)	296
*a*, *b*, *c* (Å)	18.084 (2), 7.3858 (10), 20.614 (3)
β (°)	104.725 (5)
*V* (Å^3^)	2662.9 (6)
*Z*	4
Radiation type	Mo *K*α
μ (mm^−1^)	0.09
Crystal size (mm)	0.34 × 0.19 × 0.14

Data collection	
Diffractometer	Bruker APEXII CCD
Absorption correction	Multi-scan (*SADABS*; Sheldrick, 2008 ▸)
*T*_min_, *T*_max_	0.964, 0.982
No. of measured, independent and observed [*I* > 2σ(*I*)] reflections	30340, 5064, 2847
*R*_int_	0.100
(sin θ/λ)_max_ (Å^−1^)	0.612

Refinement	
*R*[*F* ^2^ > 2σ(*F* ^2^)], *wR*(*F* ^2^), *S*	0.053, 0.159, 1.01
No. of reflections	5064
No. of parameters	331
H-atom treatment	H-atom parameters constrained
Δρ_max_, Δρ_min_ (e Å^−3^)	0.17, −0.24

Computer programs: *APEX2* and *SAINT* (Bruker, 2007 ▸), *SHELXS97* (Sheldrick, 2008 ▸), *SHELXL2014* (Sheldrick, 2015 ▸), *ORTEP-3 for Windows* (Farrugia, 2012 ▸) and *PLATON* (Spek, 2003 ▸).

## supplementary crystallographic information

### Crystal data

**Table:**

C_27_H_33_N_3_O_6_	*F*(000) = 1056
*M**_r_* = 495.56	*D*_x_ = 1.236 Mg m^−^^3^
Monoclinic, *P*2_1_/*c*	Mo *K*α radiation, λ = 0.71073 Å
*a* = 18.084 (2) Å	Cell parameters from 6177 reflections
*b* = 7.3858 (10) Å	θ = 2.3–25.6°
*c* = 20.614 (3) Å	µ = 0.09 mm^−^^1^
β = 104.725 (5)°	*T* = 296 K
*V* = 2662.9 (6) Å^3^	Block, colourless
*Z* = 4	0.34 × 0.19 × 0.14 mm

### Data collection

**Table:**

Bruker APEXII CCD diffractometer	2847 reflections with *I* > 2σ(*I*)
φ and ω scans	*R*_int_ = 0.100
Absorption correction: multi-scan (SADABS; Sheldrick, 2008)	θ_max_ = 25.8°, θ_min_ = 2.6°
*T*_min_ = 0.964, *T*_max_ = 0.982	*h* = −22→22
30340 measured reflections	*k* = −9→9
5064 independent reflections	*l* = −25→25

### Refinement

**Table:**

Refinement on *F*^2^	0 restraints
Least-squares matrix: full	Hydrogen site location: inferred from neighbouring sites
*R*[*F*^2^ > 2σ(*F*^2^)] = 0.053	H-atom parameters constrained
*wR*(*F*^2^) = 0.159	*w* = 1/[σ^2^(*F*_o_^2^) + (0.0858*P*)^2^ + 0.0315*P*] where *P* = (*F*_o_^2^ + 2*F*_c_^2^)/3
*S* = 1.01	(Δ/σ)_max_ < 0.001
5064 reflections	Δρ_max_ = 0.17 e Å^−^^3^
331 parameters	Δρ_min_ = −0.24 e Å^−^^3^

### Special details

**Table:**

Geometry. All esds (except the esd in the dihedral angle between two l.s. planes) are estimated using the full covariance matrix. The cell esds are taken into account individually in the estimation of esds in distances, angles and torsion angles; correlations between esds in cell parameters are only used when they are defined by crystal symmetry. An approximate (isotropic) treatment of cell esds is used for estimating esds involving l.s. planes.

### Fractional atomic coordinates and isotropic or equivalent isotropic displacement parameters (Å^2^)

**Table:**

	*x*	*y*	*z*	*U*_iso_*/*U*_eq_	
C1	0.24163 (12)	0.5010 (3)	0.78577 (11)	0.0352 (5)	
C2	0.13024 (12)	0.6221 (3)	0.74190 (12)	0.0375 (6)	
C3	0.16639 (12)	0.3837 (3)	0.69484 (12)	0.0369 (5)	
C4	0.36056 (12)	0.3650 (3)	0.83435 (12)	0.0394 (6)	
C5	0.41371 (13)	0.3863 (3)	0.80154 (13)	0.0474 (6)	
H5A	0.414886	0.490563	0.776511	0.057*	
C6	0.47069 (14)	0.2444 (3)	0.80498 (14)	0.0497 (7)	
C7	0.47343 (13)	0.0961 (4)	0.85502 (14)	0.0540 (7)	
H7A	0.504492	0.135622	0.898256	0.065*	
H7B	0.498106	−0.008829	0.841457	0.065*	
C8	0.39470 (13)	0.0401 (3)	0.86274 (13)	0.0457 (6)	
C9	0.34874 (15)	−0.0466 (4)	0.79846 (14)	0.0578 (7)	
H9A	0.300597	−0.087701	0.804551	0.087*	
H9B	0.376563	−0.147656	0.787322	0.087*	
H9C	0.339957	0.040625	0.762789	0.087*	
C10	0.40348 (17)	−0.0952 (4)	0.91991 (15)	0.0721 (9)	
H10A	0.353844	−0.130632	0.924139	0.108*	
H10B	0.431630	−0.040324	0.960993	0.108*	
H10C	0.430498	−0.199987	0.910640	0.108*	
C11	0.35337 (14)	0.2092 (3)	0.87747 (13)	0.0468 (6)	
H11A	0.299616	0.181148	0.871222	0.056*	
H11B	0.373899	0.242978	0.924046	0.056*	
C12	0.08311 (12)	0.8590 (3)	0.79663 (12)	0.0410 (6)	
C13	0.09560 (14)	1.0324 (3)	0.79119 (13)	0.0470 (6)	
H13A	0.102783	1.077750	0.751140	0.056*	
C14	0.09837 (14)	1.1550 (3)	0.84702 (13)	0.0491 (7)	
C15	0.07422 (14)	1.0778 (3)	0.90568 (13)	0.0478 (6)	
H15A	0.019044	1.086247	0.897016	0.057*	
H15B	0.096344	1.150036	0.945123	0.057*	
C16	0.09812 (14)	0.8803 (3)	0.92030 (13)	0.0484 (7)	
C17	0.0646 (2)	0.8065 (4)	0.97584 (16)	0.0761 (9)	
H17A	0.010036	0.819671	0.962993	0.114*	
H17B	0.085256	0.872627	1.016510	0.114*	
H17C	0.077532	0.680699	0.982980	0.114*	
C18	0.18568 (15)	0.8686 (4)	0.94248 (15)	0.0667 (8)	
H18A	0.200952	0.743947	0.948294	0.100*	
H18B	0.203935	0.931994	0.984139	0.100*	
H18C	0.206908	0.922596	0.908869	0.100*	
C19	0.06780 (14)	0.7700 (3)	0.85618 (13)	0.0465 (6)	
H19A	0.091580	0.651387	0.861718	0.056*	
H19B	0.013106	0.753238	0.848962	0.056*	
C20	0.20769 (13)	0.1412 (3)	0.63687 (11)	0.0403 (6)	
C21	0.27721 (14)	0.2252 (3)	0.62347 (13)	0.0455 (6)	
H21A	0.314724	0.246081	0.665677	0.055*	
H21B	0.263836	0.341455	0.601772	0.055*	
C22	0.31233 (14)	0.1041 (3)	0.57862 (12)	0.0449 (6)	
C23	0.26205 (16)	0.1060 (4)	0.50692 (13)	0.0592 (7)	
H23A	0.283448	0.026626	0.479581	0.089*	
H23B	0.259545	0.226814	0.489336	0.089*	
H23C	0.211536	0.065647	0.506731	0.089*	
C24	0.39175 (16)	0.1760 (4)	0.57940 (16)	0.0717 (9)	
H24A	0.414793	0.098988	0.552510	0.108*	
H24B	0.422971	0.177397	0.624662	0.108*	
H24C	0.387400	0.296629	0.561525	0.108*	
C25	0.31926 (15)	−0.0878 (3)	0.60713 (14)	0.0527 (7)	
H25A	0.334273	−0.167835	0.575397	0.063*	
H25B	0.360003	−0.089323	0.648135	0.063*	
C26	0.24829 (15)	−0.1627 (4)	0.62219 (12)	0.0497 (6)	
C27	0.19336 (14)	−0.0336 (3)	0.63526 (13)	0.0471 (6)	
H27A	0.147559	−0.074862	0.642567	0.057*	
N1	0.19417 (10)	0.6328 (2)	0.78932 (9)	0.0374 (5)	
N2	0.11151 (10)	0.5028 (3)	0.69217 (10)	0.0414 (5)	
N3	0.23251 (10)	0.3701 (2)	0.74050 (9)	0.0363 (5)	
O1	0.30770 (9)	0.5048 (2)	0.83405 (8)	0.0444 (4)	
O2	0.07577 (9)	0.7480 (2)	0.73984 (8)	0.0467 (4)	
O3	0.14991 (9)	0.2578 (2)	0.64567 (9)	0.0478 (5)	
O4	0.51468 (12)	0.2482 (3)	0.76883 (12)	0.0776 (7)	
O5	0.11854 (13)	1.3113 (2)	0.84562 (11)	0.0753 (6)	
O6	0.23846 (13)	−0.3247 (3)	0.62492 (11)	0.0795 (7)	

### Atomic displacement parameters (Å^2^)

**Table:**

	*U*^11^	*U*^22^	*U*^33^	*U*^12^	*U*^13^	*U*^23^
C1	0.0343 (12)	0.0306 (13)	0.0423 (14)	−0.0016 (10)	0.0130 (11)	−0.0023 (11)
C2	0.0371 (13)	0.0294 (12)	0.0496 (15)	0.0020 (10)	0.0180 (11)	0.0018 (11)
C3	0.0379 (13)	0.0309 (12)	0.0444 (14)	−0.0025 (10)	0.0151 (11)	−0.0044 (11)
C4	0.0342 (12)	0.0362 (13)	0.0464 (14)	0.0018 (10)	0.0078 (11)	−0.0100 (11)
C5	0.0455 (14)	0.0382 (14)	0.0624 (17)	−0.0032 (11)	0.0208 (13)	0.0002 (13)
C6	0.0394 (14)	0.0464 (15)	0.0690 (19)	−0.0048 (11)	0.0239 (13)	−0.0024 (13)
C7	0.0401 (14)	0.0529 (17)	0.0695 (19)	0.0102 (12)	0.0146 (13)	0.0069 (14)
C8	0.0415 (13)	0.0463 (15)	0.0517 (16)	0.0045 (11)	0.0161 (12)	0.0071 (13)
C9	0.0623 (17)	0.0462 (16)	0.0675 (19)	−0.0053 (13)	0.0213 (15)	−0.0026 (14)
C10	0.077 (2)	0.071 (2)	0.073 (2)	0.0139 (16)	0.0281 (17)	0.0241 (17)
C11	0.0465 (14)	0.0543 (16)	0.0436 (15)	0.0030 (12)	0.0189 (12)	−0.0003 (13)
C12	0.0340 (12)	0.0381 (14)	0.0527 (16)	0.0107 (10)	0.0145 (11)	−0.0023 (12)
C13	0.0558 (15)	0.0336 (15)	0.0558 (16)	0.0060 (11)	0.0221 (13)	0.0045 (12)
C14	0.0538 (15)	0.0309 (14)	0.0642 (18)	0.0058 (12)	0.0181 (13)	0.0019 (13)
C15	0.0491 (14)	0.0438 (15)	0.0521 (16)	0.0033 (12)	0.0156 (12)	−0.0088 (12)
C16	0.0491 (14)	0.0451 (15)	0.0558 (17)	0.0055 (11)	0.0219 (12)	0.0072 (13)
C17	0.096 (2)	0.072 (2)	0.072 (2)	0.0067 (18)	0.0417 (18)	0.0139 (17)
C18	0.0567 (17)	0.0641 (19)	0.074 (2)	0.0107 (14)	0.0062 (15)	0.0113 (16)
C19	0.0422 (13)	0.0325 (13)	0.0701 (18)	0.0037 (10)	0.0236 (13)	0.0038 (13)
C20	0.0452 (13)	0.0405 (15)	0.0355 (13)	0.0040 (11)	0.0108 (10)	−0.0076 (11)
C21	0.0522 (15)	0.0371 (14)	0.0494 (15)	−0.0047 (11)	0.0169 (12)	−0.0100 (12)
C22	0.0524 (15)	0.0406 (14)	0.0459 (15)	−0.0035 (11)	0.0202 (12)	−0.0036 (12)
C23	0.0763 (19)	0.0613 (18)	0.0448 (16)	−0.0099 (14)	0.0244 (14)	−0.0032 (14)
C24	0.0663 (19)	0.078 (2)	0.080 (2)	−0.0166 (16)	0.0360 (17)	−0.0172 (18)
C25	0.0600 (16)	0.0475 (16)	0.0561 (17)	0.0085 (13)	0.0246 (13)	−0.0044 (13)
C26	0.0709 (17)	0.0384 (16)	0.0431 (15)	0.0048 (13)	0.0204 (13)	0.0027 (12)
C27	0.0543 (15)	0.0412 (15)	0.0505 (16)	−0.0026 (12)	0.0217 (13)	−0.0038 (12)
N1	0.0361 (10)	0.0293 (10)	0.0494 (12)	0.0002 (8)	0.0156 (9)	−0.0052 (9)
N2	0.0357 (11)	0.0381 (11)	0.0511 (13)	0.0028 (8)	0.0126 (9)	−0.0059 (10)
N3	0.0383 (10)	0.0302 (10)	0.0415 (11)	0.0017 (8)	0.0120 (9)	−0.0048 (9)
O1	0.0398 (9)	0.0407 (10)	0.0508 (10)	0.0071 (7)	0.0076 (8)	−0.0120 (8)
O2	0.0436 (9)	0.0396 (9)	0.0565 (11)	0.0116 (7)	0.0117 (8)	−0.0066 (8)
O3	0.0429 (9)	0.0444 (10)	0.0539 (11)	0.0049 (7)	0.0083 (8)	−0.0169 (8)
O4	0.0715 (13)	0.0625 (13)	0.1195 (19)	0.0047 (10)	0.0626 (14)	0.0057 (12)
O5	0.1122 (17)	0.0341 (11)	0.0883 (16)	−0.0123 (11)	0.0416 (13)	−0.0049 (10)
O6	0.1165 (17)	0.0338 (12)	0.1037 (17)	0.0018 (11)	0.0566 (14)	0.0050 (11)

### Geometric parameters (Å, º)

**Table:**

C1—N1	1.312 (3)	C14—C15	1.499 (4)
C1—N3	1.325 (3)	C15—C16	1.529 (4)
C1—O1	1.347 (3)	C15—H15A	0.9700
C2—N1	1.313 (3)	C15—H15B	0.9700
C2—N2	1.329 (3)	C16—C17	1.526 (4)
C2—O2	1.348 (3)	C16—C19	1.530 (4)
C3—N2	1.317 (3)	C16—C18	1.535 (4)
C3—N3	1.324 (3)	C17—H17A	0.9600
C3—O3	1.351 (3)	C17—H17B	0.9600
C4—C5	1.318 (3)	C17—H17C	0.9600
C4—O1	1.406 (3)	C18—H18A	0.9600
C4—C11	1.480 (3)	C18—H18B	0.9600
C5—C6	1.459 (3)	C18—H18C	0.9600
C5—H5A	0.9300	C19—H19A	0.9700
C6—O4	1.221 (3)	C19—H19B	0.9700
C6—C7	1.497 (4)	C20—C27	1.316 (3)
C7—C8	1.529 (3)	C20—O3	1.401 (3)
C7—H7A	0.9700	C20—C21	1.489 (3)
C7—H7B	0.9700	C21—C22	1.535 (3)
C8—C9	1.515 (4)	C21—H21A	0.9700
C8—C10	1.522 (4)	C21—H21B	0.9700
C8—C11	1.525 (3)	C22—C23	1.526 (4)
C9—H9A	0.9600	C22—C24	1.527 (4)
C9—H9B	0.9600	C22—C25	1.528 (3)
C9—H9C	0.9600	C23—H23A	0.9600
C10—H10A	0.9600	C23—H23B	0.9600
C10—H10B	0.9600	C23—H23C	0.9600
C10—H10C	0.9600	C24—H24A	0.9600
C11—H11A	0.9700	C24—H24B	0.9600
C11—H11B	0.9700	C24—H24C	0.9600
C12—C13	1.310 (3)	C25—C26	1.501 (4)
C12—O2	1.407 (3)	C25—H25A	0.9700
C12—C19	1.479 (3)	C25—H25B	0.9700
C13—C14	1.455 (3)	C26—O6	1.213 (3)
C13—H13A	0.9300	C26—C27	1.451 (3)
C14—O5	1.213 (3)	C27—H27A	0.9300
			
N1—C1—N3	127.9 (2)	C17—C16—C18	109.4 (2)
N1—C1—O1	114.44 (19)	C15—C16—C18	109.3 (2)
N3—C1—O1	117.62 (19)	C19—C16—C18	110.1 (2)
N1—C2—N2	128.16 (19)	C16—C17—H17A	109.5
N1—C2—O2	118.7 (2)	C16—C17—H17B	109.5
N2—C2—O2	113.10 (19)	H17A—C17—H17B	109.5
N2—C3—N3	127.9 (2)	C16—C17—H17C	109.5
N2—C3—O3	114.07 (19)	H17A—C17—H17C	109.5
N3—C3—O3	117.93 (19)	H17B—C17—H17C	109.5
C5—C4—O1	119.6 (2)	C16—C18—H18A	109.5
C5—C4—C11	126.0 (2)	C16—C18—H18B	109.5
O1—C4—C11	114.2 (2)	H18A—C18—H18B	109.5
C4—C5—C6	119.2 (2)	C16—C18—H18C	109.5
C4—C5—H5A	120.4	H18A—C18—H18C	109.5
C6—C5—H5A	120.4	H18B—C18—H18C	109.5
O4—C6—C5	121.0 (2)	C12—C19—C16	112.3 (2)
O4—C6—C7	121.7 (2)	C12—C19—H19A	109.2
C5—C6—C7	117.3 (2)	C16—C19—H19A	109.2
C6—C7—C8	113.6 (2)	C12—C19—H19B	109.2
C6—C7—H7A	108.8	C16—C19—H19B	109.2
C8—C7—H7A	108.8	H19A—C19—H19B	107.9
C6—C7—H7B	108.8	C27—C20—O3	117.3 (2)
C8—C7—H7B	108.8	C27—C20—C21	125.0 (2)
H7A—C7—H7B	107.7	O3—C20—C21	117.4 (2)
C9—C8—C10	109.0 (2)	C20—C21—C22	111.55 (19)
C9—C8—C11	109.4 (2)	C20—C21—H21A	109.3
C10—C8—C11	110.1 (2)	C22—C21—H21A	109.3
C9—C8—C7	110.0 (2)	C20—C21—H21B	109.3
C10—C8—C7	110.0 (2)	C22—C21—H21B	109.3
C11—C8—C7	108.4 (2)	H21A—C21—H21B	108.0
C8—C9—H9A	109.5	C23—C22—C24	109.5 (2)
C8—C9—H9B	109.5	C23—C22—C25	110.6 (2)
H9A—C9—H9B	109.5	C24—C22—C25	109.5 (2)
C8—C9—H9C	109.5	C23—C22—C21	110.0 (2)
H9A—C9—H9C	109.5	C24—C22—C21	108.9 (2)
H9B—C9—H9C	109.5	C25—C22—C21	108.4 (2)
C8—C10—H10A	109.5	C22—C23—H23A	109.5
C8—C10—H10B	109.5	C22—C23—H23B	109.5
H10A—C10—H10B	109.5	H23A—C23—H23B	109.5
C8—C10—H10C	109.5	C22—C23—H23C	109.5
H10A—C10—H10C	109.5	H23A—C23—H23C	109.5
H10B—C10—H10C	109.5	H23B—C23—H23C	109.5
C4—C11—C8	113.3 (2)	C22—C24—H24A	109.5
C4—C11—H11A	108.9	C22—C24—H24B	109.5
C8—C11—H11A	108.9	H24A—C24—H24B	109.5
C4—C11—H11B	108.9	C22—C24—H24C	109.5
C8—C11—H11B	108.9	H24A—C24—H24C	109.5
H11A—C11—H11B	107.7	H24B—C24—H24C	109.5
C13—C12—O2	118.7 (2)	C26—C25—C22	115.5 (2)
C13—C12—C19	125.3 (2)	C26—C25—H25A	108.4
O2—C12—C19	115.7 (2)	C22—C25—H25A	108.4
C12—C13—C14	120.8 (2)	C26—C25—H25B	108.4
C12—C13—H13A	119.6	C22—C25—H25B	108.4
C14—C13—H13A	119.6	H25A—C25—H25B	107.5
O5—C14—C13	121.6 (2)	O6—C26—C27	121.6 (3)
O5—C14—C15	122.2 (2)	O6—C26—C25	121.1 (2)
C13—C14—C15	116.3 (2)	C27—C26—C25	117.3 (2)
C14—C15—C16	113.4 (2)	C20—C27—C26	120.7 (2)
C14—C15—H15A	108.9	C20—C27—H27A	119.7
C16—C15—H15A	108.9	C26—C27—H27A	119.7
C14—C15—H15B	108.9	C1—N1—C2	112.26 (19)
C16—C15—H15B	108.9	C3—N2—C2	111.66 (19)
H15A—C15—H15B	107.7	C3—N3—C1	111.95 (18)
C17—C16—C15	110.0 (2)	C1—O1—C4	117.56 (17)
C17—C16—C19	109.6 (2)	C2—O2—C12	117.53 (18)
C15—C16—C19	108.4 (2)	C3—O3—C20	119.36 (17)
			
O1—C4—C5—C6	176.9 (2)	C23—C22—C25—C26	−70.8 (3)
C11—C4—C5—C6	1.9 (4)	C24—C22—C25—C26	168.5 (2)
C4—C5—C6—O4	170.7 (3)	C21—C22—C25—C26	49.9 (3)
C4—C5—C6—C7	−10.1 (4)	C22—C25—C26—O6	157.2 (2)
O4—C6—C7—C8	−143.6 (3)	C22—C25—C26—C27	−24.7 (3)
C5—C6—C7—C8	37.1 (3)	O3—C20—C27—C26	176.2 (2)
C6—C7—C8—C9	66.4 (3)	C21—C20—C27—C26	2.5 (4)
C6—C7—C8—C10	−173.6 (2)	O6—C26—C27—C20	175.0 (3)
C6—C7—C8—C11	−53.2 (3)	C25—C26—C27—C20	−3.2 (4)
C5—C4—C11—C8	−20.7 (3)	N3—C1—N1—C2	2.1 (3)
O1—C4—C11—C8	164.03 (19)	O1—C1—N1—C2	−179.09 (18)
C9—C8—C11—C4	−75.6 (3)	N2—C2—N1—C1	−2.9 (3)
C10—C8—C11—C4	164.6 (2)	O2—C2—N1—C1	179.37 (19)
C7—C8—C11—C4	44.3 (3)	N3—C3—N2—C2	2.2 (3)
O2—C12—C13—C14	175.7 (2)	O3—C3—N2—C2	179.37 (19)
C19—C12—C13—C14	2.5 (4)	N1—C2—N2—C3	1.0 (3)
C12—C13—C14—O5	171.3 (2)	O2—C2—N2—C3	178.84 (19)
C12—C13—C14—C15	−9.3 (4)	N2—C3—N3—C1	−2.8 (3)
O5—C14—C15—C16	−144.1 (3)	O3—C3—N3—C1	−179.92 (19)
C13—C14—C15—C16	36.5 (3)	N1—C1—N3—C3	0.4 (3)
C14—C15—C16—C17	−174.3 (2)	O1—C1—N3—C3	−178.33 (19)
C14—C15—C16—C19	−54.5 (3)	N1—C1—O1—C4	178.46 (19)
C14—C15—C16—C18	65.5 (3)	N3—C1—O1—C4	−2.6 (3)
C13—C12—C19—C16	−22.7 (3)	C5—C4—O1—C1	91.5 (3)
O2—C12—C19—C16	163.90 (18)	C11—C4—O1—C1	−92.9 (2)
C17—C16—C19—C12	166.6 (2)	N1—C2—O2—C12	−13.8 (3)
C15—C16—C19—C12	46.6 (3)	N2—C2—O2—C12	168.13 (19)
C18—C16—C19—C12	−73.0 (3)	C13—C12—O2—C2	113.9 (2)
C27—C20—C21—C22	25.3 (3)	C19—C12—O2—C2	−72.3 (2)
O3—C20—C21—C22	−148.4 (2)	N2—C3—O3—C20	170.2 (2)
C20—C21—C22—C23	72.3 (3)	N3—C3—O3—C20	−12.3 (3)
C20—C21—C22—C24	−167.7 (2)	C27—C20—O3—C3	127.7 (2)
C20—C21—C22—C25	−48.7 (3)	C21—C20—O3—C3	−58.1 (3)

### Hydrogen-bond geometry (Å, º)

**Table:**

*D*—H···*A*	*D*—H	H···*A*	*D*···*A*	*D*—H···*A*
C5—H5*A*···O4^i^	0.93	2.59	3.447 (3)	153
C19—H19*A*···O5^ii^	0.97	2.60	3.532 (3)	162
C21—H21*B*···O6^iii^	0.97	2.57	3.399 (3)	143

Symmetry codes: (i) −*x*+1, *y*+1/2, −*z*+3/2; (ii) *x*, *y*−1, *z*; (iii) *x*, *y*+1, *z*.

## References

[bb1] Borisova, K. K., Nikitina, E. V., Novikov, R. A., Khrustalev, V. N., Dorovatovskii, P. V., Zubavichus, Y. V., Kuznetsov, M. L., Zaytsev, V. P., Varlamov, A. V. & Zubkov, F. I. (2018). *Chem. Commun.* **54**, 2850–2853. 10.1039/c7cc09466c 29393318

[bb2] Bruker (2007). *APEX2* and *SAINT*. Bruker AXS Inc., Madison, Wisconsin, USA.

[bb3] Farrugia, L. J. (2012). *J. Appl. Cryst.* **45**, 849–854.

[bb4] Gurbanov, A. V., Mahmoudi, G., Guedes da Silva, M. F. C., Zubkov, F. I., Mahmudov, K. T. & Pombeiro, A. J. L. (2018). *Inorg. Chim. Acta*, **471**, 130–136.

[bb5] Gurbanov, A. V., Mahmudov, K. T., Kopylovich, M. N., Guedes da Silva, F. M., Sutradhar, M., Guseinov, F. I., Zubkov, F. I., Maharramov, A. M. & Pombeiro, A. J. L. (2017*a*). *Dyes Pigments*, **138**, 107–111.

[bb6] Gurbanov, A. V., Mahmudov, K. T., Sutradhar, M., Guedes da Silva, F. C., Mahmudov, T. A., Guseinov, F. I., Zubkov, F. I., Maharramov, A. M. & Pombeiro, A. J. L. (2017*b*). *J. Organomet. Chem.* **834**, 22–27.

[bb7] Hathwar, V. R., Sist, M., Jørgensen, M. R. V., Mamakhel, A. H., Wang, X., Hoffmann, C. M., Sugimoto, K., Overgaard, J. & Iversen, B. B. (2015). *IUCrJ*, **2**, 563–574. 10.1107/S2052252515012130 PMC4547824 26306198

[bb8] Jlassi, R., Ribeiro, A. P. C., Alegria, E. C. B. A., Naïli, H., Tiago, G. A. O., Rüffer, T., Lang, H., Zubkov, F. I., Pombeiro, A. J. L. & Rekik, W. (2018). *Inorg. Chim. Acta*, **471**, 658–663.

[bb9] Ledenyova, I. V., Falaleev, A. V., Shikhaliev, Kh. S., Ryzhkova, E. A. & Zubkov, F. I. (2018). *Russ. J. Gen. Chem.* **88**, 73–79.

[bb10] Ma, Z., Gurbanov, A. V., Maharramov, A. M., Guseinov, F. I., Kopylovich, M. N., Zubkov, F. I., Mahmudov, K. T. & Pombeiro, A. J. L. (2017*a*). *J. Mol. Catal. A Chem.* **426**, 526–533.

[bb11] Ma, Z., Gurbanov, A. V., Sutradhar, M., Kopylovich, M. N., Mahmudov, K. T., Maharramov, A. M., Guseinov, F. I., Zubkov, F. I. & Pombeiro, A. J. L. (2017*b*). *J. Mol. Catal. A Chem.* **428**, 17–23.

[bb12] Mahmudov, K. T., Kopylovich, M. N., Guedes da Silva, M. F. C. & Pombeiro, A. J. L. (2017). *Coord. Chem. Rev.* **345**, 54–72.

[bb13] Mahmudov, K. T. & Pombeiro, A. J. L. (2016). *Chem. Eur. J.* **22**, 16356–16398. 10.1002/chem.201601766 27492126

[bb14] McKinnon, J. J., Jayatilaka, D. & Spackman, M. A. (2007). *Chem. Commun.* pp. 3814–3816. 10.1039/b704980c 18217656

[bb15] Nasirova, D. K., Malkova, A. V., Polyanskii, K. B., Yankina, K. Y., Amoyaw, P. N.-A., Kolesnik, I. A., Kletskov, A. V., Godovikov, I. A., Nikitina, E. V. & Zubkov, F. I. (2017). *Tetrahedron Lett.* **58**, 4384–4387.

[bb16] Sheldrick, G. M. (2008). *Acta Cryst.* A**64**, 112–122. 10.1107/S0108767307043930 18156677

[bb17] Sheldrick, G. M. (2015). *Acta Cryst.* C**71**, 3–8.

[bb18] Spek, A. L. (2003). *J. Appl. Cryst.* **36**, 7–13.

[bb19] Sughanya, V. & Sureshbabu, N. (2012). *Acta Cryst.* E**68**, o2875–o2876. 10.1107/S1600536812037853 PMC3470230 23125674

[bb20] Vandyshev, D. Y., Shikhaliev, K. S., Potapov, A. Y., Krysin, M. Y., Zubkov, F. I. & Sapronova, L. V. (2017). *Beilstein J. Org. Chem.* **13**, 2561–2568. 10.3762/bjoc.13.252 PMC5727784 29259665

[bb21] Yang, X.-H., Zhou, Y.-H., Zhang, M. & Hu, L.-H. (2011). *Acta Cryst.* E**67**, o492. 10.1107/S1600536811002698 PMC3051563 21523148

[bb22] Yusifov, N. N., Ismayilov, V. M., Sadigova, N. D., Kopylovich, M. N. & Mahmudov, K. T. (2013). *Mendeleev Commun.* **23**, 292–293.

